# Interactions between Biliverdin, Oxidative Damage, and Spleen Morphology after Simulated Aggressive Encounters in Veiled Chameleons

**DOI:** 10.1371/journal.pone.0138007

**Published:** 2015-09-14

**Authors:** Michael W. Butler, Russell A. Ligon

**Affiliations:** 1 Department of Biology, Lafayette College, Easton, Pennsylvania, United States of America; 2 School of Life Sciences, Arizona State University, Tempe, Arizona, United States of America; University of Arkansas for Medical Sciences; College of Pharmacy, UNITED STATES

## Abstract

Stressors frequently increase oxidative damage–unless organisms simultaneously mount effective antioxidant responses. One putative mitigative mechanism is the use of biliverdin, an antioxidant produced in the spleen during erythrocyte degradation. We hypothesized that both wild and captive-bred male veiled chameleons (*Chamaeleo calyptratus*), which are highly aggressive to conspecifics, would respond to agonistic displays with increased levels of oxidative damage, but that increased levels of biliverdin would limit this increase. We found that even just visual exposure to a potential combatant resulted in decreased body mass during the subsequent 48-hour period, but that hematocrit, biliverdin concentration in the bile, relative spleen size, and oxidative damage in plasma, liver, and spleen were unaffected. Contrary to our predictions, we found that individuals with *smaller* spleens exhibited greater decreases in hematocrit and higher bile biliverdin concentrations, suggesting a revision to the idea of spleen-dependent erythrocyte processing. Interestingly, individuals with larger spleens had reduced oxidative damage in both the liver and spleen, demonstrating the spleen’s importance in modulating oxidative damage. We also uncovered differences in spleen size and oxidative damage between wild and captive-bred chameleons, highlighting environmentally dependent differences in oxidative physiology. Lastly, we found no relationship between oxidative damage and biliverdin concentration, calling into question biliverdin’s antioxidant role in this species.

## Introduction

Oxidative damage occurs when biomolecules such as DNA, lipids, and proteins are oxidized [1], resulting in decreased functionality of basic physiological processes [2,3]. The extent of such oxidative damage is driven by variation in numerous physiological factors, including energetic demands [4], hormone titers (e.g., glucocorticoids [4]), and antioxidant defenses [5]. Thus, when organisms are confronted with changes to their metabolic needs or fluctuations in hormone levels, they may concurrently be challenged with increased rates of oxidative damage. The interdependency between key physiological processes and concomitant increases in oxidative damage suggests that oxidative damage may serve as an important life history constraint in many ecological settings [6–8], and may even be a ubiquitous mechanism driving life history trade-offs [9,10].

Variation in oxidative damage among individuals may be partially explained by the organism’s exposure to stressors. Stressors result in the up-regulation of the hypothalamus-pituitary-adrenal (HPA) axis, which regulates traits as diverse as nutrient metabolism, immune function, and behavior. However, HPA upregulation can also result in immunosuppression, glycogenolysis, and protein catabolism (see [11] for a review). Because many of these effects involve changes to metabolic processes, one of the physiological consequences of stressors may be oxidative stress; indeed, experimental increases in glucocorticoid levels can increase oxidative damage [1]. However, the relative importance of oxidative damage to an animal’s fitness depends upon the specific nature of oxidative damage, which can vary by the types of biomolecules that are damaged [6] and the degree of damage among different organs [12]. Such inter-organ variation in oxidative damage may be driven by differences in organ-specific metabolic processes [13] or antioxidant defenses [14]. Consequently, a stressor that results in an increase in oxidative damage may have organ-specific effects, thereby exerting costs specific to that organ.

Within this framework, the spleen may be an especially important organ in mediating stress-induced oxidative damage. In mice, intravenous infusion of epinephrine, a hormone associated with stress, decreases spleen size–possibly via changes in blood flow [15]. Conversely, administration of antioxidants increases the size of the spleen in mice [16] and chickens [17] via unidentified mechanisms. Oxidative damage to erythrocytes also appears to have an effect on spleen size; when erythrocytes exhibit greater amounts of oxidative damage, the spleen increases in size [18]. Thus, while the spleen’s immunological functions are undoubtedly critical, its role in mediating oxidative damage in response to stressors may have an underappreciated importance. For example, the spleen’s ability to change size in response to stressors and antioxidants may allow an individual to minimize oxidative damage in response to varying pro-oxidant or antioxidant levels.

While there is wealth of literature regarding how animals use both exogenous and endogenous antioxidants to minimize oxidative stress [19], one underexplored mechanism of antioxidant defense is the spleen’s production of the endogenous antioxidant, biliverdin [20]. Biliverdin is synthesized in the spleen from free heme molecules that are by-products of erythrocyte destruction [21]. After production in the spleen, biliverdin is transferred to the liver where it is secreted into the bile (at least in fish and reptiles; [21]). Because stress-induced oxidative damage frequently manifests via erythrocyte destruction [22,23], hematocrit levels should decrease in stressed animals and, consequently, biliverdin levels should increase. Such biliverdin-dependent increases in antioxidant capacity may then buffer increases in oxidative damage for at least a brief period, which would explain the seeming delayed relationship between glucocorticoids and oxidative damage (see Fig 3 in [1]).

To investigate how biologically relevant stressors affect oxidative damage in relation to biliverdin production and spleen size, we exposed male veiled chameleons (*Chamaeleo calyptratus*) to either non-physical agonistic or sham encounters. As part of their territorial response, veiled chameleons undergo dramatic physiological color changes and behavioral responses when exposed to conspecific males [24,25], and thus presumably undergo substantial physiological shifts prior to any physical encounter. Because agonistic intraspecific encounters cause physiological stress responses in a wide variety of vertebrate species [26–29], aggression may also increase oxidative stress in animals involved in aggressive encounters. Considering that aggression can play a pivotal role in the behavioral ecology of an organism [30], it also seems likely that animals whose life-history includes frequent or high-intensity aggressive interactions may have evolved means to ameliorate aggression-associated oxidative damage via concomitant elevations of antioxidant defense(s) ([31,32], but see [33]).

We hypothesized that aggressive interactions between male veiled chameleons, even in the absence of physical contact, would result in oxidative damage and resultant red blood cell breakdown. Because oxidative damage can result in erythrocyte destruction (hemolysis) which can increase spleen size [34,35], we predicted that individuals exposed to aggressive interactions would have larger spleens and greater levels of biliverdin in the bile as a consequence of the destruction of oxidatively damaged erythrocytes within the subsequent 48 hours, a time period based on work with both rats [22] and birds [23], due to the lack of such studies with squamates. Additionally, we hypothesized that biliverdin may consequently act as an antioxidant in such a context, thus minimizing further oxidative damage. Behavioral or physiological processes that typically generate reactive oxygen species may not always result in increased levels of oxidative damage if antioxidant defenses (either enzymatic or molecular) are concomitantly increased [36]. Thus, under this hypothesis, we predicted that animals exposed to agonistic encounters would exhibit elevated biliverdin levels, but minimal or non-existent increases in oxidative damage, leading to a negative correlation between biliverdin levels and oxidative damage. However, if biliverdin is not a biologically relevant antioxidant in this species, then not only will oxidative damage due to agonistic interactions be substantial, but there will be no relationship between biliverdin and oxidative damage. Further, because prior experience with stressors can affect stress response [37], and stress responses may be repeatable throughout the lifetime of the individual [38], we predicted that individuals bred in captivity would have a reduced stress response resulting in less oxidative damage, while individuals from a wild, introduced population in Florida that had presumably dealt with a greater amount of stressors would have a greater stress response, resulting in greater oxidative damage (*sensu* [28]).

## Materials and Methods

### Behavioral treatment

Veiled chameleons are large, territorial lizards native to the southwestern region of the Arabian peninsula [39,40]. As part of their territorial response, male veiled chameleons undergo dramatic color changes and display behaviors when exposed to conspecific males [24,25,40]. Because exposure to conspecifics mimics natural encounters between territorial animals [40], such encounters represent an ecologically relevant stressor for veiled chameleons (RA Ligon, *unpub data*). Therefore, we exposed adult male veiled chameleons in our treatment group (N = 10) to one another via non-physical agonistic encounters to assess the relationship between biliverdin and biologically relevant, behaviorally induced stress. Specifically, we placed the cages of individuals in the agonistic treatment next to one another in a manner that facilitated visual access but prevented physical access. Each chameleon in the treatment group was exposed to three different opponents over the course of a six hour treatment period, facing the cage of each opponent for two hours. Based on the reproductive biology of the closely related common chameleon *Chamaeleo chamaeleon*, exposure to numerous conspecific competitors on a given day is highly likely [41]. In contrast, control animals (N = 10) experienced sham encounters wherein they faced empty cages that were rotated every two hours to control for the disturbance caused by cage rotation. Thus, chameleons in both groups experienced similar levels of cage manipulation but markedly different levels of conspecific exposure. Animals were acquired from both a private breeder and a wild population in Florida, and origin was balanced across treatments (Treatment group: 5 wild, 5 captive; Control group: 4 wild, 6 captive). All chameleons were similarly sized (mean: 192 g, standard deviation: 40 g; see S1 Dataset). After encounters, both groups of chameleons continued to be misted multiple times daily but were not given any additional food during the subsequent 48-hour period to minimize human disturbance.

### Blood collection

Prior to any behavioral exposures (see above), we drew 0.5 ml of whole blood from each chameleon from the caudal vein using heparinized syringes. Forty-eight hours after the beginning of the experimental exposure to opponents (or control cages), we collected another blood sample from each animal (this time from the ventricle of anesthetized chameleons, see below). Micro-capillary tubes were used to collect a small volume of blood from the Eppendorf tubes and these capillary tubes were used to assess hematocrit levels from each animal. All samples were kept on ice until centrifugation, at which point plasma was drawn off and frozen at -80°C.

### Tissue collection

Forty-eight hours following the behavioral trials, chameleons were anesthetized via inhalation of isoflurane, an anesthetic that has minimal effects on plasma antioxidant levels [42], until they became un-responsive. We then collected a blood sample from the heart and decapitated the animals before commencing tissue collection, which ensured death as approved by the ASU Institutional Animal Care and Use Committee (protocol 13-1308R). We did not pith the brain so as to preserve the brain for possible future studies. For this study, from each chameleon we collected the intact spleen, approximately 0.5 g of liver, and bile from the gallbladder. These tissue samples were immediately placed on ice until being frozen at -80°C.

### Oxidative damage assessment

We quantified oxidative damage using a thiobarbituric acid reactive substances kit (OxiSelect STA-330; Cell Biolabs, Inc., CA), which measures the end products of lipid peroxidation (malondialdehyde; MDA). While this assay does not capture all metrics of systemic oxidative damage because it lacks the ability to quantify oxidative damage to proteins and DNA, it does capture those markers of oxidative damage that are most pertinent to this study, namely the damage done to lipids in the erythrocyte membranes that would result in the destruction of damaged erythrocytes. We followed the kit instructions, with the following emendations. We homogenized approximately 50 mg of liver (wet mass; range 53 to 56 mg) and between 3.8 and 90 mg (wet mass) of spleen in 1 mL phosphate-buffered saline in 1X BHT (kit component). This range in spleen sample weights was due to the large variance in spleen total weight, from 3.8 mg to 760 mg (mean = 91 mg, median = 24.3 mg). All liver and spleen MDA values were then corrected for sample mass. We used the butanol extraction method for all plasma, liver, and spleen MDA samples. Some plasma samples were beyond the linear range of the standard curve, but subsequent dilution and reanalysis (several weeks later) resulted in values below the detectable limit, suggesting that the lag in processing time resulted in sample degradation; these samples were omitted from the study. All liver and spleen samples fell within the linear range.

### Biliverdin quantification

Because there are currently no methodologies for quantifying biliverdin in plasma in non-pathological samples [43], and biliverdin can have incredibly low recovery rates even in biliverdin-rich tissues [44], we were limited to quantifying biliverdin solely in bile, for which recovery is greater than 90% [45]. Bile samples (N = 17; 3 samples [see S1 Dataset for details] did not have enough material) were thawed and vortexed prior to analysis; several samples (N = 5) had small, unidentified particulates suspended within the bile that precipitated out during centrifugation (see below). Following [45], we mixed 30 μl of bile with 170 μl of 5:6 HCl 3N:acetonitrile, vortexed briefly, and centrifuged the sample for 3 min at 3420 g (6,000 rpm). The supernatant was analyzed via high performance liquid chromatography using an Alltech Apollo C18 column (150mm, ID = 4.6 mm), with a flow rate of 1.0 ml/min, and an Agilent (Santa Clara CA) 1100 series with UV-vis spectrometry detection. The solvent gradient consisted of an initial mobile phase of methanol (25%) and ammonium acetate 75% (1.0 M, pH adjusted to 5.16), followed by an 8 min linear change to 95% methanol, 5% ammonium acetate, then 2 min at these conditions, and finally 8 min at 25% methanol, 75% ammonium acetate prior to the initiation of the next sample. The column was maintained at 70°C, and absorbance was measured at 376 nm. Absorbance values were within the linear range of a standard curve of a known standard (biliverdin hydrocholoride, B655-9, Frontier Scientific, Logan, UT).

### Statistics

Spleen-Somatic Index was calculated as spleen weight (g)/body weight (g) * 10^6^ (*sensu* [46]). Not all variables were normally distributed, so we log-transformed Spleen-Somatic Index and the MDA measures of spleen and pre-treatment plasma samples to meet the assumption of normality. Hematocrit values could not be transformed to achieve normality, but the residuals from these analyses were normally distributed unless otherwise noted. We used analysis of variance to test for effects of treatment and origin (captive-bred vs. wild) on biliverdin concentration in the bile and MDA levels in multiple tissues, analysis of covariance to test for the effect of treatment and origin on change in body mass with pre-treatment body mass as a covariate, and a mixed model with individual as a random effect to test for treatment-based differences in hematocrit both before and after treatment.

To test for relationships between spleen size and physiological states, we performed simple linear regressions with average body mass (both pre- and post-trial body mass), biliverdin concentration in the bile, change in hematocrit during the course of the experiment, and MDA in all tissues as dependent variables and the log of Spleen-Somatic Index as the independent variable. Because we were explicitly interested in biliverdin’s role in mitigating oxidative damage, we also ran simple linear regressions between biliverdin concentration in the bile and MDA of each tissue. While some metrics are clearly correlated (e.g., post-trial MDA and change in MDA), we elected to analyze all variables so as ensure we did not miss any potentially interesting findings in this initial investigation relating biliverdin and oxidative damage. Effect sizes are calculated as Hedges’s *g*, which is commonly referred to as Cohen’s *d* in the literature [47], and although caution should be exercised when using benchmark values to interpret the magnitude of an effect size, *d* values greater than 0.8 have traditionally been viewed as large [47]. All statistics were performed with SAS 9.3 (Cary, NC).

## Results

### Treatment and Origin Effects

**Fig 1:**
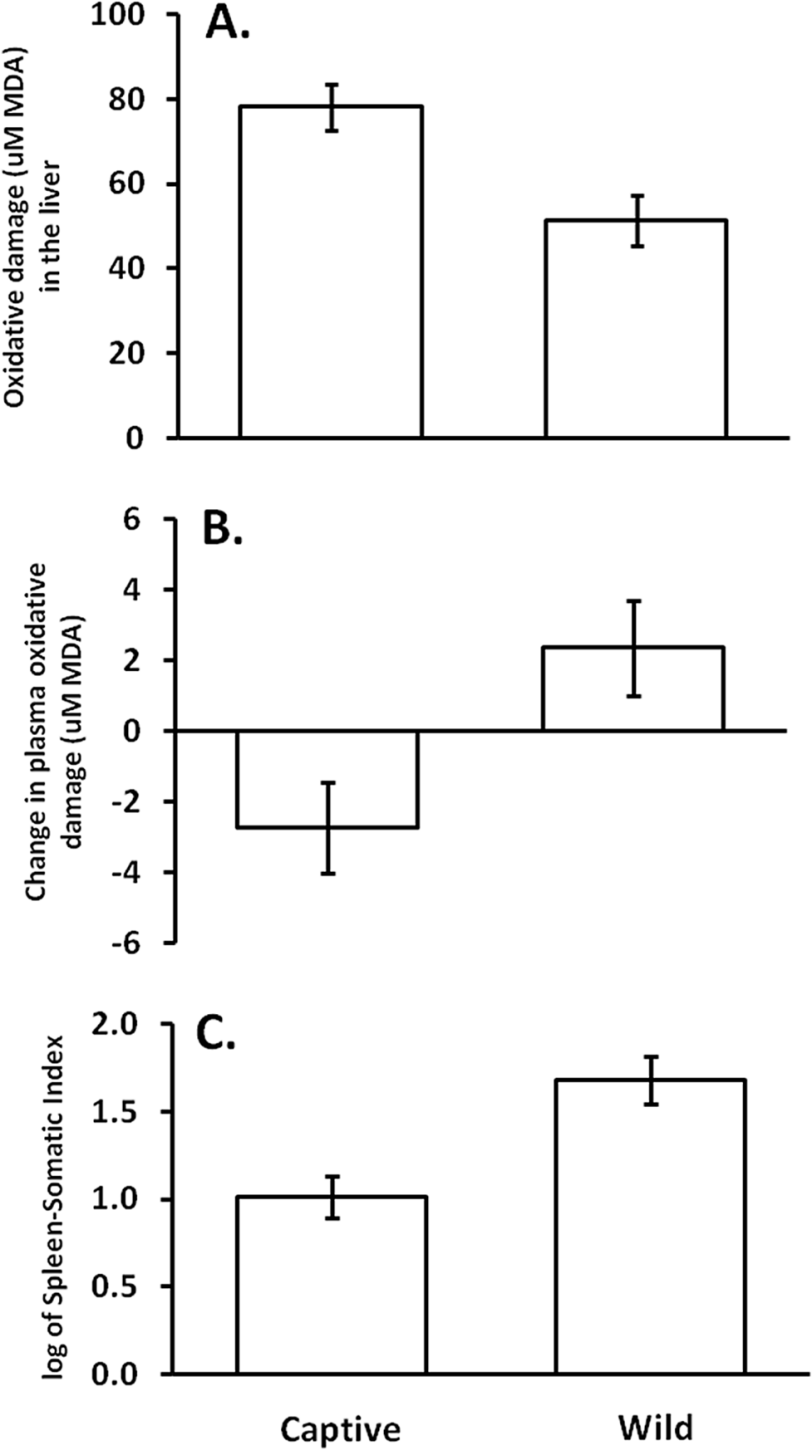
Differences between wild and captive-raised chameleons. Chameleons that were raised in captivity had (A) larger amounts of oxidative damage in the liver, but (B) reduced the circulating amount of oxidative damage in plasma over the 48-hour time period relative to wild chameleons, regardless of treatment. Captive-bred chameleons also had (C) relatively smaller spleens. LSMeans are shown, ± standard error.

Chameleons that were exposed to conspecifics lost more body mass (least square mean = -8.48g, SE = ± 1.2g) than control animals (-3.69g ± 1.27g) over the duration of the experiment (F_1,15_ = 7.77, *P* = 0.0138, effect size = 1.40). However, conspecific exposure did not affect biliverdin levels in the bile, Spleen-Somatic Index, MDA levels in spleen, liver, or plasma, or change in plasma MDA levels over the duration of the experiment (Table 1). Furthermore, neither treatment, time point, nor their interaction affected hematocrit levels (all F_1,33_ < 1.10, all *P* > 0.3). However, individual origin affected multiple metrics, with chameleons collected from the wild population having reduced MDA levels in the liver, an increase of plasma MDA during the 48-hour trial, and larger Spleen-Somatic Indices (Table 1; Fig 1).

**Table 1:** Effect of treatment on multiple metrics of chameleon physiology.

Dependent variable	Independent variable	d.f.	F	*P*	Effect size
Change in mass	**Treatment**	**1,15**	**7.77**	**0.0138**	**1.40**
	Origin	1,15	3.28	0.0900	0.77
Biliverdin	Treatment	1,14	0.10	0.7519	0.17
	Origin	1,14	0.00	0.9910	0.06
Liver MDA	Treatment	1,17	0.02	0.8787	0.18
	**Origin**	**1,17**	**11.16**	**0.0039**	**1.56**
Log Spleen MDA	Treatment	1,17	1.92	0.1833	0.50
	Origin	1,17	3.69	0.0715	0.79
Log Plasma MDA (pre-encounter)	Treatment	1,13	0.58	0.4585	0.38
	Origin	1,13	2.34	0.1503	0.79
Plasma MDA (post-encounter)	Treatment	1,9	1.76	0.2174	0.73
	Origin	1,9	0.51	0.4928	0.28
Change in plasma MDA over 48 hrs	Treatment	1,9	0.17	0.6860	0.41
	**Origin**	**1,9**	**7.52**	**0.0228**	**1.72**
Spleen-Somatic Index	Treatment	1,17	0.18	0.6759	0.27
	**Origin**	**1,17**	**13.81**	**0.0017**	**1.74**

Chameleons that were visually exposed to other chameleons lost significantly more mass than those exposed to an empty control arena. Treatment did not affect any other metric. Significant relationships (*P* < 0.05 and effect sizes greater than 1) are indicated in bold type.

To explicitly test the role biliverdin might have in modulating oxidative damage, we included biliverdin concentration as a covariate for analyses with MDA concentration as a dependent variable. In all cases, neither treatment nor biliverdin concentration predicted MDA concentration in any tissue, although origin continued to explain variation in liver MDA levels (Table 2).

**Table 2:** Oxidative damage in multiple tissues was not affected by treatment when biliverdin concentration in the bile was included as a covariate.

Dependent variable	Independent variable	d.f.	F	*P*
Liver MDA	Treatment	1,13	0.63	0.44
	**Origin**	**1,13**	**5.06**	**0.04**
	Biliverdin concentration	1,13	0.01	0.92
Log Spleen MDA	Treatment	1,13	0.55	0.47
	Origin	1,13	1.63	0.22
	Biliverdin concentration	1,13	0.80	0.39
Log Plasma MDA (pre-encounter)	Treatment	1,9	0.23	0.65
	Origin	1,9	1.41	0.26
	Biliverdin concentration	1,9	0.12	0.74
Log Plasma MDA (post-encounter)	Treatment	1,5	0.49	0.52
	Origin	1,5	1.06	0.35
	Biliverdin concentration	1,5	0.80	0.41
Change in plasma MDA over 48 hrs	Treatment	1,5	0.12	0.74
	Origin	1,5	3.20	0.13
	Biliverdin concentration	1,5	0.31	0.60

Significant relationships (*P* < 0.05) are indicated in bold type.

### Spleen-Somatic Index

**Fig 2:**
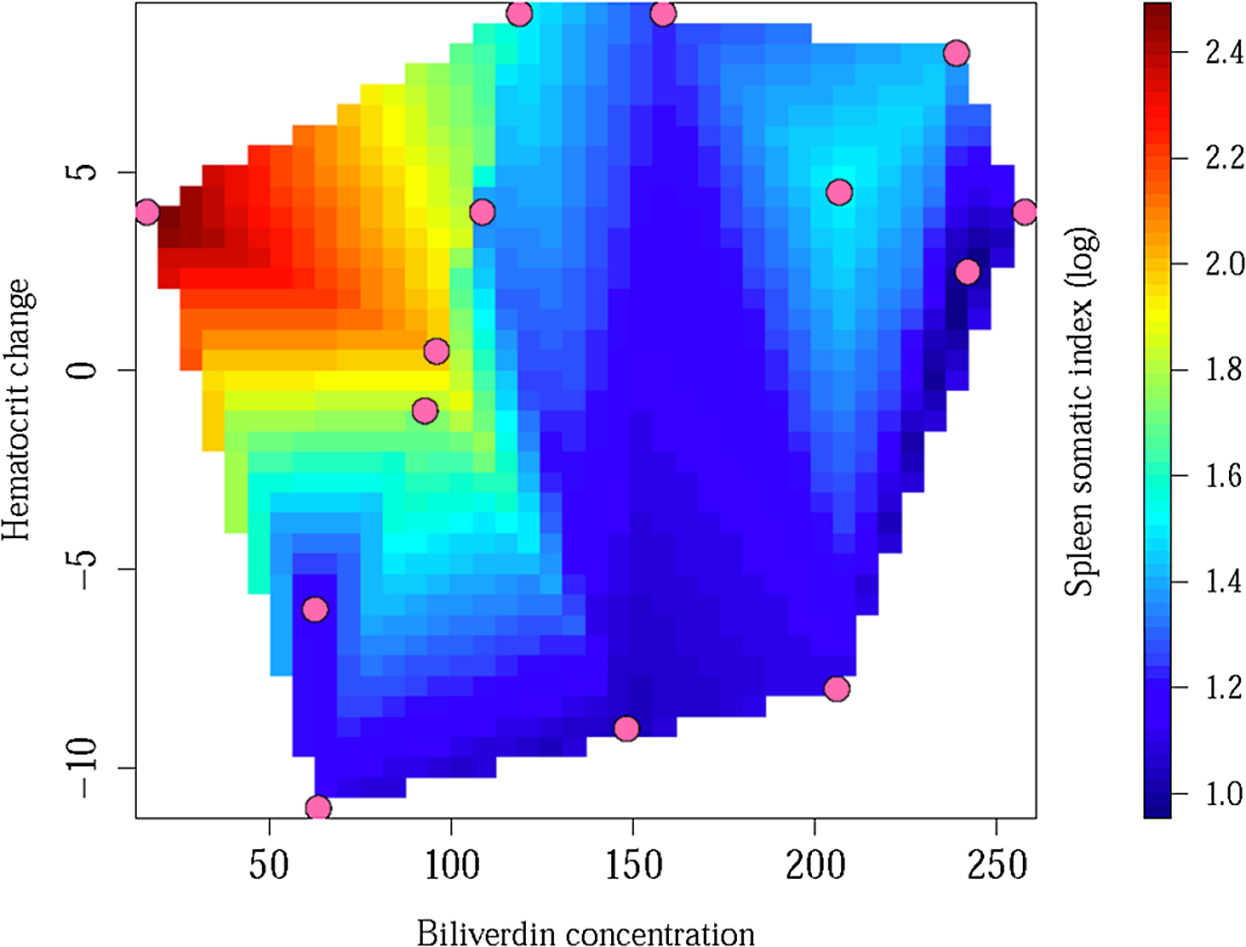
Variation in spleen size, hematocrit, and biliverdin concentration. Relative spleen size (indicated by the color legend on the right) was related to changes in hematocrit (y-axis) and biliverdin concentrations in bile (x-axis). Specifically, relatively larger spleens were associated with increases in hematocrit and lower concentrations of biliverdin in bile, regardless of treatment. Together, biliverdin concentration and hematocrit change explained 45% of the variation in relative spleen size (Adj-R^2^ = 0.45).

Neither change in hematocrit over the course of the experiment nor biliverdin concentration in the bile independently predicted Spleen-Somatic Index (Table 3). However, because we were explicitly interested in biliverdin production as erythrocytes were degraded in the spleen, we also ran a model with both biliverdin concentration and change in hematocrit as independent variables and Spleen-Somatic Index as a dependent variable (these two independent variables were not intercorrelated; F_1,12_ = 0.99, *P* = 0.34). We found that chameleons with greater concentrations of biliverdin in the bile (F_1,11_ = 9.99, *P* = 0.009) and larger drops in hematocrit (F_1,11_ = 6.05, *P* = 0.032) had smaller spleens, counter to our predictions (Fig 2). However, because origin could explain some of the variation in Spleen-Somatic Index, we ran this same model with origin as an independent variable. Wild chameleons had larger SSIs than captive-raised chameleons (F_1,10_ = 12.07, *P* = 0.0060; Fig 1C), but even after accounting for this variation, chameleons with greater concentrations of biliverdin in the bile had smaller spleens (F_1,10_ = 15.93, *P* = 0.0026), but there was no longer any relationship between change in hematocrit and spleen size (F_1,10_ = 1.22, *P* = 0.30).

**Table 3:** Log of Spleen-Somatic Index (SSI) was not significantly predicted by change in hematocrit over the duration of the experiment or in biliverdin concentration in the bile.

Independent variable	Parameter estimate	Standard error	d.f.	F	*P*
Change in hematocrit	-0.0053	0.0199	1,15	0.07	0.79
Biliverdin concentration	-0.00219	0.00123	1,15	3.17	0.095

**Fig 3:**
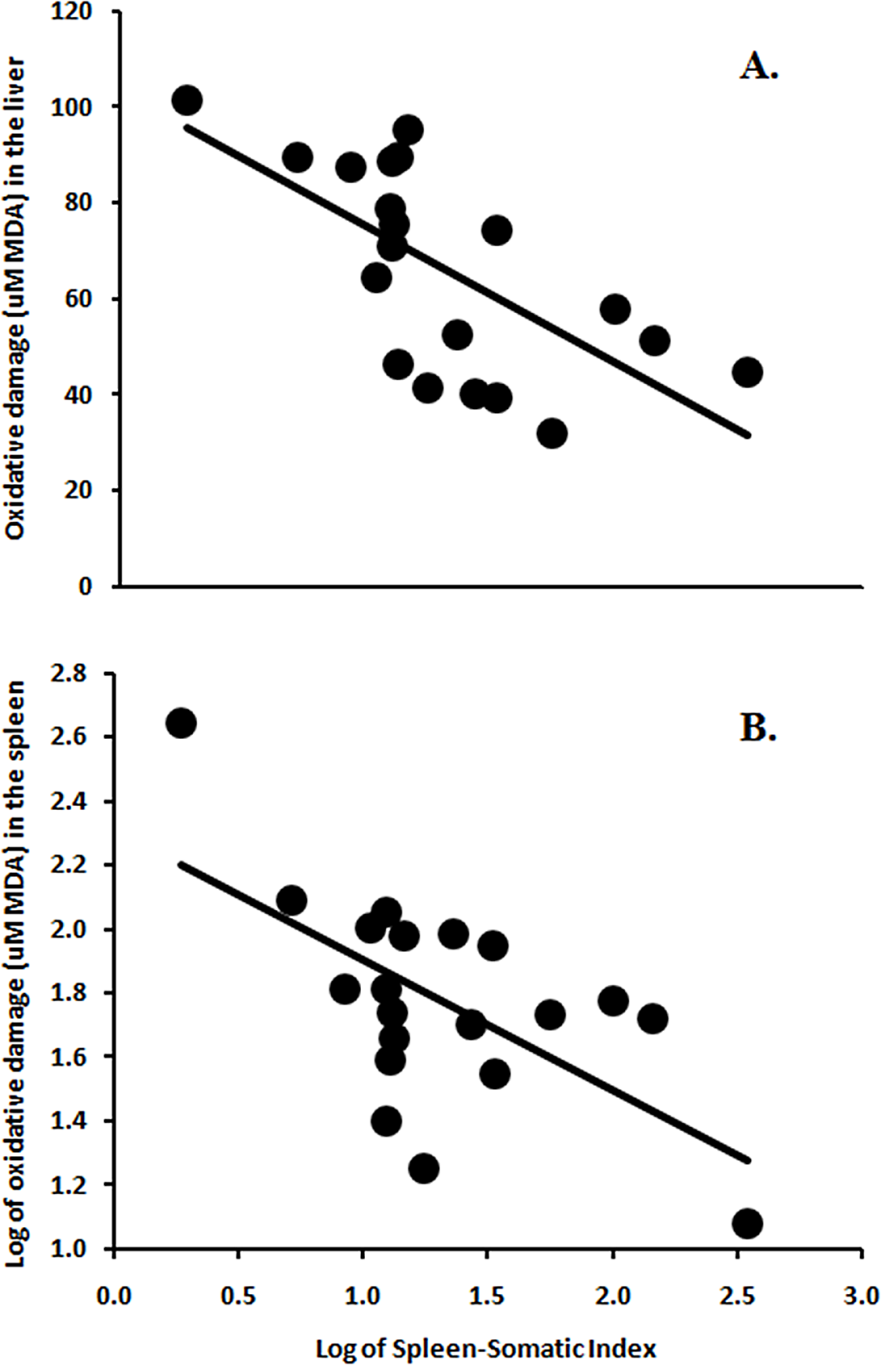
Spleen size and oxidative damage in liver and spleen. Regardless of treatment, chameleons with relatively larger spleens had lower levels of oxidative damage (as assessed by production of MDA) in both liver (A) and spleen (B) tissues.

Spleen-Somatic Index was negatively correlated to MDA concentration in both liver (F_1,18_ = 14.18, *P* = 0.001; Fig 3A) and in spleen (F_1,18_ = 11.42, *P* = 0.003; Fig 3B) tissue; chameleons with relatively larger spleens had less oxidative damage in the liver and spleen. When origin was included as an independent variable, these relationships became less robust (Liver: F_1,17_ = 3.54, *P* = 0.077; Spleen: F_1,17_ = 6.86, *P* = 0.018). There was no significant relationship between Spleen-Somatic Index and plasma MDA concentration at the start (F_1,14_ = 2.46, *P* = 0.14), at the end (F_1,10_ = 0.69, *P* = 0.43), or the change over the course of the experiment (F_1,10_ = 0.91, *P* = 0.36). Inclusion of origin as an independent variable did not qualitatively affect these relationships (all *P* > 0.05).

### Oxidative Damage and Biliverdin Concentration

Because there were no treatment effects on oxidative damage or biliverdin concentration, we ran simple linear regressions between these two terms for all chameleons regardless of treatment. There were no significant relationships between biliverdin concentration in the bile and MDA concentration in any tissue (Table 4). Including origin as an independent variable did not qualitatively affect these relationships (all *P* > 0.1).

**Table 4:** Oxidative damage was not significantly related to biliverdin concentration in the bile.

Dependent variable	Parameter estimate	Standard Error	d.f.	F	*P*
Liver MDA	0.0146	0.06738	1,15	0.05	0.83
Log Spleen MDA	0.00075256	0.000864	1,15	0.76	0.40
Log Plasma MDA (pre-encounter)	0.00096179	0.0015	1,11	0.41	0.53
Log Plasma MDA (post-encounter)	0.03263	0.0179	1,7	3.32	0.11
Change in plasma MDA	-0.00086768	0.02108	1,7	0.00	0.97

## Discussion

Veiled chameleons are highly aggressive toward conspecifics, and even in the absence of physical contact, we found that these behaviors have significant costs. Chameleons exposed to non-physical agonistic encounters lost approximately 5 more grams over the subsequent 48 hr period than those that were exposed to empty cages, an increase of approximately 42%. This finding mirrors the costs of aggressive interactions in American bison (*Bison bison;* [48]), where individuals engaged in more aggressive interactions lost significantly more mass than individuals who fought less often. Likewise, both male fallow deer (*Dama dama*; [49]) and black grouse (*Tetrao tetrix*; [50]) that engaged in more fights lost more mass. However, despite the apparent metabolic costs associated with agonistic interactions, including non-physical interactions such as the ones in this study, we found no influence of conspecific exposure on any additional physiological parameter.

Agonistic encounters induce cortiocosterone increases in multiple species [51], and while we did not explicitly quantify circulating corticosterone levels for logistical reasons, the observed decrease in body mass corresponds with the predicted effects of increased stress in general [52] and corticosterone in particular [53]. Thus, our results demonstrate that even non-physical agonistic encounters, in this case simple visual access to a competitor, are sufficient to result in demonstrable changes to phenotype. However, we detected no other effects of these agonistic encounters. Despite previously documented general relationships between increased stress and increases in reactive oxygen metabolites [54], we did not detect any treatment-based differences in lipid peroxidation (MDA concentration) in any tissue, relative spleen size, or biliverdin levels. We suggest two explanations for these unexpected findings. First, the time course of our study may have precluded our detection of an effect. We picked the 48-hr time point because work with chukars (*Alectoris chukar*) suggested that captivity stress reduces hematocrit for 1–3 days post-stressor [23] and because rats exposed to an immobilization stressor have fewer red blood cells and increased MDA concentrations in plasma 48-hr after the stressor [22]. However, because there has been no previous work investigating these parameters in squamates, it is possible our time frame for measuring physiological response to conspecific exposure missed a critical window due to taxonomic differences between chameleons and chukars or rats, such as the major physiological differences between endotherms and ectotherms. Second, due to the frequency and intensity with which chameleons engage in aggressive behaviors [41], it is possible that chameleons have evolved physiological mechanisms to mitigate stress-induced damage initiated by agonistic encounters. If so, then veiled chameleons would be an appropriate study species for future work investigating oxidative stress and changes in glucocorticoid concentration in response to both natural and unnatural stressors.

Despite biliverdin's reported antioxidant capability in other species [55], we found no evidence to suggest that biliverdin acts as an antioxidant *in vivo* in veiled chameleons. However, the framework of our hypothesis depended upon detecting treatment-based differences in lipid peroxidation in the liver, spleen, or plasma, or documenting a change in biliverdin concentration in the bile. Because no such treatment-based differences existed for either the induction of oxidative damage or a change in biliverdin concentration, biliverdin’s role as an antioxidant *in vivo* remains unclear. Despite interest in understanding biliverdin’s role as an antioxidant, the vast majority of investigations use mammals, which are a sub-optimal model for this question. Mammals produce large amounts of the enzyme biliverdin reductase, which reduces biliverdin to bilirubin, another molecule with antioxidant properties *in vitro* [20]. Such enzymatic conversions would confound any putative findings of biliverdin acting as an antioxidant in mammals, as bilirubin could be the molecule acting as an antioxidant. However, for many birds, non-avian reptiles, and some fish, biliverdin is the predominant end product of heme degradation [56], and thus the antioxidant nature of biliverdin can be more directly investigated in future studies using these taxa, rather than in mammalian taxa that readily convert biliverdin to bilirubin.

There are several explanations regarding the discrepancy between biliverdin’s demonstrated antioxidant properties *in vitro* and the lack of an antioxidant role in our system. The first is that biliverdin is hydrophilic at physiological pH [57], and MDA is predominately a marker of lipid-based oxidative damage. While both hydrophilic and lipophilic antioxidants frequently interact to affect a single trait [58], it is possible that biliverdin does not offer protection to many lipids. The second is that biliverdin truly has no antioxidant role *in vivo*, and our data are consistent with this hypothesis. However, several other hypotheses are also supported by our data. For example, it is possible that our treatment did induce oxidative damage, but chameleons responded by increasing their antioxidant defenses, either via biliverdin production or some other means, thus minimizing or eliminating any detectable change in oxidative damage 48 hours later. To resolve this question, we would need to either manipulate circulating biliverdin levels or oxidative damage more directly, or collect samples from chameleons at repeated time intervals, which is currently methodologically unfeasible considering the current terminal, bile-dependent methodology required to quantify biliverdin in any non-pathological samples [43,44].

Despite the lack of evidence regarding an antioxidant role for biliverdin, we uncovered interesting relationships between biliverdin concentration in the bile, relative spleen size, and hematocrit levels. Specifically, we found an interactive relationship between changes in hematocrit and bile biliverdin concentrations − in direct contrast to our predictions. Originally, we predicted that chameleons’ spleens would become enlarged as they processed a greater number of damaged red blood cells that would, in turn, lead to increased biliverdin concentration in the bile. However, we found that relative spleen size was greater in individuals with *increasing* hematocrit and *low* biliverdin concentration in the bile. Even after accounting for origin-dependent differences in relative spleen size, the negative relationship between relative spleen size and bile biliverdin concentration persisted. This result stands in contrast to much of the literature, which documents evidence that spleen size increases as erythrocytes are sequestered [18] and hematocrit decreases [35], and that increased levels of biliverdin are associated with destruction of erythrocytes [59,60]. However, these previous experiments examined genetic variants, incidence of disease, or hyperbiliverdinemia, and thus may have documented patterns not typical of more common processes. Our contrasting findings, in conjunction with the evidence that our treatment either did not induce oxidative damage or that it had already been ameliorated, suggest that the pattern we uncovered may reflect more natural variation in these metrics. Within this framework, spleen size may be minimally plastic and individuals with relatively smaller spleens may simply be more efficient at producing biliverdin and in removing damaged red blood cells, thus allowing them to perform those physiological roles with a reduced somatic investment. Though our experiment was not designed to test these relationships explicitly, future work that examines links between spleen size, biliverdin concentration in bile or other tissues, and changes in hematocrit in unmanipulated animals could explore these potential relationships in greater detail.

We uncovered robust relationships between relative spleen size and levels of oxidative damage in both the spleen and liver, but not plasma. These relationships fit proposed mechanistic pathways, and positive relationships between spleen size and antioxidants [16,17] and negative relationships between spleen size and oxidative damage [61] have previously been established. However, our findings underscore two main points. First, while sampling plasma has the benefit of allowing the assessment of oxidative damage at multiple time points or permitting non-terminal work, we found that plasma does not necessarily reflect variation in other tissues. Second, we corroborated previous work showing that the spleen may be a particularly important organ in managing levels of oxidative damage (e.g., [62]). Interestingly, this relationship existed not only between relative spleen size and oxidative damage in the spleen, but also in oxidative damage in the liver, suggesting a general linkage between oxidative damage and spleen size.

The importance of spleen size in the physiological status of veiled chameleons is underscored by the differences detected between individuals from the wild versus captive-bred source populations. Captive-bred chameleons had relatively smaller spleens, suggesting that either genetic differences or organizational effects of rearing environment (e.g., reduced frequency of high-intensity stressors or increased frequency of low-intensity stressors) drive variation in spleen size between wild and captive-bred chameleons. Such differences in relative spleen size may be driven by population-dependent differences in sex hormones [63] or glucocorticoid [64] levels, although we lack the data to objectively assess this prediction. We also uncovered origin-based differences in oxidative damage, with reduced oxidative damage in the plasma of captive-bred animals over the 48 hour experimental period. This finding is consistent with the previously mentioned possibility that smaller spleens, such as those belonging to captive-bred individuals, perform optimally while simultaneously minimizing somatic investment. However, captive-bred chameleons also showed higher oxidative damage in liver tissue compared to wild chameleons (Fig 1), counter to our predictions. The precise mechanisms driving these patterns are unclear, but previous work with two genetically distinct lines of mice that differ in aggression has shown that such population-level differences in oxidative damage manifest in mammals [33], suggesting the need for further work identifying physiological mechanisms driving intraspecific variation in both aggression and oxidative damage. Our results also further highlight the difference between oxidative damage patterns in plasma versus other tissues, highlighting the value of sampling multiple tissues for oxidative damage whenever feasible.

In sum, we found that even in the absence of physical contact, agonistic encounters can have negative effects (e.g., decreased body mass) in potential combatants. However, these stressors, which are common in veiled chameleons, failed to induce any other detectable effects in hematocrit, oxidative damage, relative spleen size, or biliverdin production, suggesting that these species are generally robust to such ecologically relevant stressors, or that actual fighting may be necessary to affect these parameters. We also found that relative spleen size is correlated with biliverdin concentration in the bile, but opposite to the direction that we predicted. This suggests that either veiled chameleons have a particularly unique plasticity in spleen size, or that natural variation in spleen size represents an opposite pattern to plasticity in spleen size. Lastly, we found that relative spleen size is negatively correlated with oxidative damage in both the spleen and liver, highlighting the potentially important role the spleen has in managing systemic levels of oxidative damage.

## Supporting Information

S1 Dataset Complete dataset pertaining to this manuscript. (XLSX)
